# Alzheimer's Disease and Glaucoma: Imaging the Biomarkers of Neurodegenerative Disease

**DOI:** 10.4061/2010/793931

**Published:** 2011-01-05

**Authors:** Denise A. Valenti

**Affiliations:** Vision Care, 62 Forest Avenue Quincy, MA 02169, USA

## Abstract

Imaging through the visual system in Alzheimer's disease, with the technology currently in widespread use for the diagnosis and management of eye disease such as glaucoma and macular degeneration, is proving to be promising. In vivo cross-section imaging during an annual comprehensive eye exam has been available for a decade for glaucoma and macular degeneration, and this same imaging, using Optical Coherence Tomography, has been demonstrated to show deficits specific to AD and mild cognitive impairment. These deficits are in the form of nerve fiber layer tissue drop out in the retina and optic nerve. The retrograde loss of nerve fiber layer tissue in the retina and optic nerve may be an early biomarker of AD, and these deficits in the nerve fiber layer of the retina and optic nerve may be the earliest sign of AD, even prior to damage to the hippocampal region that impacts memory.

## 1. Introduction

Biomarkers for the early diagnosis, management, and treatment of Alzheimer's disease (AD) are important. In vivo cross-section imaging during an annual comprehensive eye exam with a dilated pupil has been available for a decade for glaucoma and macular degeneration, and this same imaging, using Optical Coherence Tomography (OCT), has been demonstrated to show deficits specific to AD [1–7] and mild cognitive impairment (MCI) [2]. These deficits are in the form of nerve fiber layer tissue drop out in the retina and optic nerve. The retrograde loss of nerve fiber layer tissue in the retina and optic nerve may be an early biomarker of AD, and these deficits in the nerve fiber layer of the retina and optic nerve may be the earliest sign of AD, even prior to damage to the hippocampal region that impacts memory. The OCT technology used to identify nerve fiber layer defects in AD reported in the literature is an older version often referred to as Time Domain-OCT. Time Domain-OCT (Time-OCT) only resolved the retina to approximately ten microns, whereas newer clinical OCT imaging systems High Definition/Resolution/Fourier/Spectral Domain-OCT (HDR-OCT) can resolve the retina to five microns or less, acquire images faster, reregister retinal location, and does not require the dilation of the pupils of the eye. Faster acquisition is important for patients with cognitive impairment such as AD. The ability to reregister exact location enables more accurate followup and longitudinal study. 

OCT is similar to ultrasound, but instead of measuring echoed sound waves, it measures the echoed time delays of a reflected light beam. As the beam is scanned, there is a cross-sectional image generated. The OCT used for ocular imaging has a fiber optic delivery system coupled with a slit-biomicroscope. The patient sits with their head positioned in a chin rest and the experience is similar to having a photograph taken of the eye. This type of imaging can easily be incorporated into annual eye exams for those with AD.

## 2. History

The first published reports of imaging in vivo retinal tissue with OCT were in 1993 [8, 9], and the first reports of OCT use showing significant deficits in the eyes of participants with AD were in 2001 [5]. The technology has evolved considerably since these early works were published. OCT technology and its application in the eye had origins in the Department of Electrical Engineering and Computer Science at Massachusetts Institute of Technology where James G. Fujimoto, Ph.D., a professor in computer sciences and electrical engineering along with his students had collaborated with Carmen A. Puliafito, MD who at the time was affiliated with the Massachusetts Eye and Ear Infirmary (MEEI) [10]. Another student working with Dr. Fujimoto contributed with the idea to generalize axial scanned images to transverse images [10] and published the precursor images of ex vivo retina [11] and these are considered to be the first illustration of OCT use. Joel S. Schuman, MD was a glaucoma fellow at MEEI in 1989 and was doing work in the laboratory of Dr. Puliafito. He eventually worked closely with the New England Eye Center at Tufts Medical School where over 10,000 patients were imaged with the initial prototype [10]. Clinical studies took place between 1994 and 1995 and Carl Zeiss Meditec made available the first commercial instrument in 1996, the Stratus-OCT [10]. Optovue, Carl Zeiss Meditec, Heidelberg Engineering, Topcon Medical Systems, and Bioptigen are some of the other companies that have developed imaging technology using the HDR-OCT systems.

All publications to date, using OCT technology in the study of AD, have used the Stratus-OCT from Carl Zeiss Meditec which was the Time-OCT. The parameters are longitudinal axial resolution <10 microns in tissue, transverse resolution 20 microns in tissue, scan speed 400 A scans per second, longitudinal depth 2 mm in tissue, and an optical source of superluminescent diode at 840 nanometers [12]. The newer HDR-OCT from Carl Zeiss Meditec; the Cirrus/HD-OCT has improvements that will make the technology more effective in AD. Among the improvements is the ability to reregister the images assuring more precise, repeat measurements for longitudinal studies. The parameters are longitudinal axial resolution <5 microns in tissue, transverse resolution 15 microns in tissue, scan speed 27,000 scans per second, longitudinal depth 2 mm in tissue, and an optical source of superluminescent diode at 840 nanometers [13]. 6 shows Stratus-OCT images of the nerve fiber layer in a normal age control participant that was age matched to the AD participant, a participant with AD and a participant with both glaucoma and Parkinson's disease (not age matched). 

While it is likely that HDR-OCT technology will become an integral part of the diagnosis and management of AD, differentiating the optic nerve fiber losses and retinal changes from common age-related ocular disease, and in particular glaucoma will remain clinically demanding. The two diseases, AD and glaucoma, impact the same visual pathways early in their disease processes. Additionally both diseases can result in subtle changes in the functional aspects of vision. 

The OCT imaging picks up the deficits related to AD as structural drop out due to retrograde degeneration from the brain tissue in visual processing areas such as the visual association area, Brodmann 19. Further, the degeneration in the visual association area may be one of the earliest brain tissues damaged by AD, even before the memory/hippocampal areas. That neurodegenerative pathology of AD originates in the visual association area is indicated in the studies by McKee and colleagues of autopsied brains in the Framingham Heart Study [14]. McKee and colleagues found that in the visual association-Brodmann 19 area of over 50% of those that had been classified as being cognitively normal, having no memory impairments, there were findings related to AD such as beta amyloid and neurofibrillary tangles. Similar structural findings were commonly associated with Alzheimer's disease. In some of the cognitively normal cases, there was no AD-related pathology in the hippocampus area which processes memory, but there was AD related pathology in the Visual Association-Brodman 19 [14].

Losses in visual function attributed to AD have many aspects in common with the functional losses in glaucoma. Both diseases show structural changes early in their course in the visual cortex [15, 16] and lateral geniculate nucleus [17–19]. The ability to detect motion is reduced in AD [20, 21]; there are visual field defects [22, 23]; contrast sensitivity deficits in the lower spatial frequencies have been reported in participants with AD [24]. Glaucoma also impacts lower spatial frequencies in contrast sensitivity and produces visual field defects [25].

## 3. Glaucoma and AD [26]

There is a more aggressive destruction of structure and function when patients have both AD and glaucoma [27]. Glaucoma is a painless and insidious disease process and often goes undetected. Glaucoma impacts up to three million Americans, but only one half of those with glaucoma are aware they have it. Glaucoma is responsible for 9–12% of blindness in the United States. Glaucoma is the leading cause of blindness among African Americans and Hispanics. Those over the age of 60 of any race are at greater risk of developing glaucoma and it is estimated that 8% of those over the age of 80 have elevated IOP. Glaucoma and AD affect the magnocellular visual processing. The magnocellular-mediated visual functions are those that are not easily assessed during conventional eye examinations such as motion processing and contrast sensitivity function. There are losses in the magnocellular layers of the lateral geniculate nucleus in glaucoma [17, 18]. The lateral geniculate nucleus neurons early on in the glaucoma disease process show significant shrinkage and are observed even before nerve fiber loss in the optic nerve with conventional direct viewing methods [28, 29]. HDR-OCT likely identifies these early losses. A single case study reported in the literature by Gupta and colleagues [15] regarding changes in the visual cortex in human glaucoma indicated reductions in autopsied tissue in the area below the calcarine sulcus compared to normal control. This region of change corresponded to an area of superior visual field loss that had been identified in the participant prior to death. Structural defects specific to the magnocellular visual pathway have also been identified in individuals with AD even in brain areas devoid of plaques and neurofibrillary tangles. The magnocellular visual pathway shows signs of individual cell drop out in the primary visual cortex of AD individuals [16]. Neurofibrillary tangles and amyloid plaques have been identified in the lingual and cuneal gyri of participants with AD, and these correlate with the incidence of functional visual field loss in AD participants [22]. The magnocellular layers in the lateral geniculate nucleus have been shown to have plaques associated with AD [19]. The visual association area processes signals from the magnocellular visual pathway. 

Glaucoma and AD have disruptions in circadian function [30]. “Sundowning” may be caused by disruptions in the circadian rhythm of AD patients. Sundowning is a general term describing symptoms such as sleep disturbance, nocturnal delirium, disorientation at the onset of darkness, night-time activity, and agitation [31]. Disturbances in the circadian rhythm occur in AD patients, with rates ranging from 12% [32] to 25% [33]. AD is associated with changes in the suprachiasmatic nucleus [34, 35]. 

The eye's intraocular pressure follows a circadian rhythm with increases in intraocular pressure at night [36–39]. There are reports that found greater fluctuation and range in intraocular pressure in individuals with glaucoma compared to healthy control individuals [39, 40]. Studies by Liu and colleagues [38] found that the larger the diurnal variation and fluctuation of intraocular pressure, the greater the risk for structural damage due to glaucoma. Intraocular fluctuation may not be a risk factor, but actually an early symptom of glaucoma, as the disease impacts the optic nerve and the axons leading to brain structures processing circadian function. Light has a strong influence on the circadian system and the timing and duration of exposure to daylight can impact circadian phases, melatonin suppression, physiological responses, and alertness [41]. A previously unknown photoreceptor that was dissimilar to both rod cells and cone cells was identified by Berson and colleagues [42]. These cells are retinal ganglion cells that directly innervate the suprachiasmatic nucleus and are reactive to light even when all synaptic input from rod cells and cone cells is blocked. Damage anywhere along the visual pathways that process functions related to the circadian retinal cells has potential to disrupt circadian rhythms, and these structural deficits will result in further reduction of the nerve fiber layers in the retina as measured by OCT. This would be the case whether the site of origin is in the brain such as in AD or in the optic nerve as in glaucoma. 

**Figure 1:**
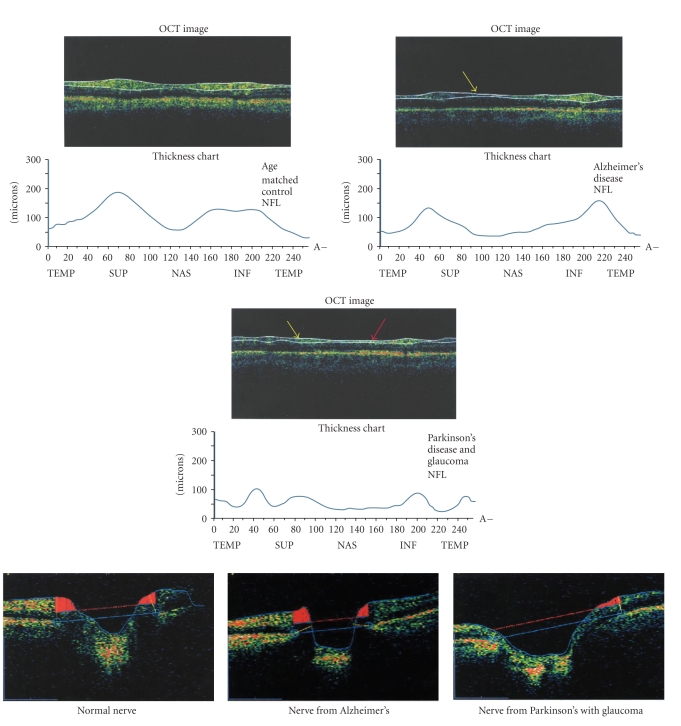
The top three images depict the nerve fiber layer (NFL) generated by the older technology; Stratus-OCT. The *y* axis is NFL thickness in microns and the *x* axis is the circumference of the optic nerve in degrees; TEMP = temporal, SUP = superior, NAS = nasal and INF = inferior. None of the participants had a significant refractive error. The top left figure is from a 73 year old control participant with no known risk factors for glaucoma; Caucasian and age matched to the AD participant. The NFL had an average thickness of 101 microns. The top right image was obtained from a 72 year old participant with no known risk factors for glaucoma; Caucasian, with AD. The NFL had an average thickness of 75 microns. The middle image was from a Caucasian participant, with no known risk factors for glaucoma; that had both Parkinson's disease and glaucoma. The average NFL thickness was 58 microns. The yellow arrows show regions of thinning hypothesized to be due to retrograde neurologic degeneration. The red arrow indicates areas of thinning attributed to glaucoma. The bottom three images are cross sections of the optic nerve. Sections of red indicate the nerve fiber layer. The bottom left image is of the control participant, the bottom middle image is of the AD participant and the bottom right image is of the participant with both Parkinson's disease and glaucoma.

Studies have identified a higher incidence of glaucoma among those residing in nursing homes. One such study found a higher rate of glaucoma among 112 residents with AD compared to 774 residents without AD. The diagnosis of glaucoma was based on visual field defects or optic nerve cupping observed during eye examinations. While there may be field defects in AD, there is generally no optic nerve cupping. Optic nerve cupping is a biomarker related to glaucoma. The rate of glaucoma was 25.9% for the AD patients and 5.2% for the control nursing home group [43]. The optic nerve may be less resistant to elevated intraocular pressure levels with AD. A record review in a large glaucoma clinic identified seven patients with elevated intraocular pressures or ocular hypertension that initially had normal visual fields and normal optic discs and had also been diagnosed within one year of also having AD. Among these patients, there was a more glaucomatous optic neuropathy which was much more rapid compared to other glaucoma patients [27]. This has also been reported to be the case for Parkinson's disease also [44]. Figure 1 shows the nerve fiber layer deficits identified with Time-OCT in a participant that had both glaucoma and Parkinson's disease. Figure 1 also shows the same participants optic nerve, demonstrating both nerve fiber loss and optic nerve cupping.

A study found a higher rate of glaucoma among AD patients compared to non-AD patients. In their study of 172 patients with AD in Japan compared to 176 age matched controls, Tamura and colleagues [45] found those with AD had a rate of glaucoma of 23.8% compared to only 9.9% of the age-matched controls having glaucoma. If there are two neurodegenerative processes such as glaucoma and AD affecting one of the major relay centers for visual function, it is easy to appreciate that the loss in function can be substantial. 

As AD progresses causing substantial reductions in brain volumes, there may be additional impact on the visual system creating abnormal pressure gradients at the back of the eye along the nerve. This can cause stress and damage to the lamina cribrosa, the supportive tissue to the optic nerve. When there is a lower pressure on the posterior nerve such as was found in a study of cerebrospinal fluid pressure by Berdahl and Allingham, there is a significantly higher rate of glaucoma [46]. In their study, the average cerebrospinal fluid pressure was 33% lower in those with glaucoma compared to those without a diagnosis of glaucoma. Morgan and colleagues theorize that when the cerebrospinal fluid pressure is low, there is an influence on subarachnoid space. Because of the space location posterior to the globe and nerve and its pressure relationships to intracranial, retrolaminar and translaminar tissue it is as if there is damage much like would occur with an elevation of pressure inside the eye [47]. There is increasing evidence that low cerebrospinal fluid pressure causes glaucoma like damage [48–50]. Wostyn and colleagues have had long standing theories regarding changes in cranial pressure and the relationship of the stress of change to neurodegenerative diseases including AD and glaucoma [51], and the recent findings related to reduced cerebrospinal fluid and glaucoma are supportive of this. Wostyn reports in a study that a subgroup of AD participants had low cerebrospinal fluid pressures and he attributed this to brain atrophy. He further theorizes, as did Morgan and colleagues, that this produces an abnormal high translamina pressure difference and this is what leads to the higher incidence of glaucoma among those with AD [52]. 

The relationship between AD and glaucoma remains poorly understood. Published studies look at more advanced dementia in already diagnosed AD and residents in nursing homes with AD. To date there are no published studies investigating glaucoma in Mild Cognitive Impairment or in mild AD. This still leaves the possibility that the two diseases are unrelated, but because they affect the same axonal structures and visual pathways the result is an expression of the vision losses related to glaucoma at an earlier stage of the disease. It is estimated that thirty to fifty percent of ganglion cells and fibers in the visual pathway are damaged by glaucoma before the disease is detected by conventional visual field testing of the peripheral vision [53, 54]. As AD destroys visual pathway fibers, the result may simply be an earlier expression of the functional losses in glaucoma because there are fewer neural reserves.

## 4. Ocular AD-Related Histopathology [26, 30]

AD damages the lateral geniculate nucleus [19] and glaucoma damages the lateral geniculate nucleus early in the respective disease processes [18]. Damage secondary to AD also occurs in the visual association cortex and other higher cortical areas, the primary visual cortex [16, 55], and visual association areas [14]. Additional studies have demonstrated deficits in the retinocalcarine pathway [56–60].

Loss of retinal ganglion cells has been identified by several groups. Such losses will be better characterized by imaging with HDR-OCT. A study by Blanks and colleagues [61] studied 16 individuals with AD with an age range of 76–93. There were 19 normal individuals with an age range from 55–91. They found that within the ganglion cell layer, the cells having the largest diameter may have been preferentially affected in AD. The cells in the magnocellular layer are larger diameter cells. 

 Sadun and Bassi [60] analyzed 3 eyes from AD individuals ranging in age from 76–89 years. There was degeneration of the retinal ganglion cells. They found axonal degeneration upon examining the retrobulbar optic nerves. The degeneration was reported to be more pronounced the more posterior the nerve. This indicated the retinal ganglion cell loss may be secondary to retrograde axonal degeneration. Hinton and colleagues [57] analyzed the optic nerves of 10 individuals with AD and 10 age-matched normal individuals ages 76–89 years. This study found eight of the ten nerves obtained from participants with AD compared to the controls were significantly different in fiber count compared to the normal individuals. The level of AD severity or duration of illness was not documented. The described findings are in contrast to the findings of Curcio and colleagues [56] who examined four AD eyes with an age range of 67–86 years as well as the eyes from four age-matched normal individuals. The individuals with AD had the disease for at least four years and had severe dementia. Curcio and colleagues did not find difference between the eyes from participants with AD compared to the age-matched control eyes. Davies and colleagues [62] studied 9 AD and 7 normal eyes and found no histopathologic evidence of differences in the retinal ganglion cells between individuals with AD and age-matched normal individuals. With imaging using UHR-OCT, we can now measure in vivo, without discomfort, but still obtain detailed imaging of structures that parallel common histologic methods, but eliminates artifacts related to tissue movement, cell displacement, cell shrinkage or nerve fiber dissipation. The UHR-OCT allows for repeat measurements of the same participant over time with image reregistration. The older OCT technology had limits in reregistering location.

## 5. Retinal Photographs and AD [26, 30]

Using photographs, Hedges and colleagues [59] studied the retinal nerve layer of 26 participants in various stages of AD and 23 age-matched controls with ages of both groups ranging from 52 to 93. They found a greater amount of nerve fiber loss in the individuals with AD compared to age-matched normal individuals. There was a trend towards increasing nerve fiber layer abnormalities with progression and duration of the disease. However, it was not statistically significant.

In the retinal photographs of 22 AD participants and 24 normal age-matched participants, Tsai and colleagues [63] found that five of the AD participants had nerve fiber loss compared to one of the control participants. The photographic method showed that there was a significant correlation in degree of segment optic nerve pallor and the duration of AD. Trends were identified in the segment pallor and the mean Alzheimer's disease Assessment Scale (cognitive, no cognitive and total). Duration and severity of AD probably impact optic nerve findings. Conventional photographic studies that include only early-onset AD may result in conclusions that the optic nerve is not impacted (and therefore no change in the ganglion cells) because the sample studied has not had the progression of the disease to the point where the ganglion cells have been impacted. However, in the study using OCT looking at both AD and MCI, Pacquet and colleagues found no statistical difference between AD and MCI indicating that the OCT imaging may be more sensitive to early disease than any previous histologic or photographic method.

## 6. Optic Nerve Fiber Imaging and AD with Stratus-OCT [26]

Parisi and colleagues [5] used the older version of Time-OCT to assess the optic nerve fiber layer thickness in 17 AD participants and 14 age-matched control participants and found a significant reduction in nerve fiber thickness in the AD individuals compared to the age-matched control participants. The age range was from 63 to 77. The study included participants with mild severity of cognitive impairment. OCT has the ability to measure the thickness of the macula. 

Early reports using imaging technologies other than OCT failed to show deficits in the nerve fiber layer associated with AD. Scanning laser polarimetry (GDx Nerve Fiber Analyzer) assesses the nerve fiber layer by measuring changes in the polarization due to the bi-refringent properties of the nerve fiber layer. Once aligned, the image is acquired in 0.7 seconds. Kergoat and colleagues [64] used this technique and found no differences in the optic nerve fiber layer in 30 individuals with early-stage AD, compared to age-matched normal individuals. The same group failed to find nerve fiber layer deficits using yet another imaging technology [65]. The technology, scanning laser tomography (Heidelberg Retinal Tomograph-HRT), uses confocal scanning lasers to provide real-time three-dimensional images and measurements of the optic disc and surrounding area. Parameters that can be measured include cup area, disc area, cup volume, rim volume, cup/disc area ratio, and rim dim/disc area. Once aligned, images are obtained in 1.5 seconds. 

Iseri and colleagues used OCT imaging technology to measure macular volume [4] and found that of 28 eyes tested in group of Alzheimer's participants there was significant thinning of the macula compared to 30 eyes tested from a group of control participants. Iseri and colleagues also measured thinning of the retinal nerve fiber layer in those with AD compared to age-matched control participants and that both the macular thinning and nerve fiber layer thinning were correlated with the severity of cognitive function. Berisha and colleagues [66] have reported the only study to have looked at all three groups; glaucoma, AD, and age-matched control participants. The group of glaucoma participants had an average age of 71, AD participants had an average age of 76, and the age matched control participants had an average age of 72. They found significant differences between the groups in the superior quadrant thickness of the nerve fiber layer when measured with OCT and abnormal cup to disc ratios in only the glaucoma group. The glaucoma participants had an average of 106 mm in the superior optic nerve fiber layer; the AD participants had an average of 90 mm, and the control participants an average of 115 mm. This study demonstrated that the deficits identified with OCT are unique to AD and are not undiagnosed ocular pathology such as glaucoma. Superior nerve fiber layer deficits were also identified in those with AD compared to normal age-matched control participants in a study by Lu and colleagues [6]. This study looked at 22 participants with AD and 22 age-matched control participants. This study, unlike other studies using OCT, found an increase in the cup to disc ratio of participants with AD compared to age-matched control participants as well as inferior nerve layer deficits compared to the age matched control participants. While not an OCT study, Danesh-Meyers and colleagues used the ocular imaging technology using scanning laser Tomography, HRT [67], and also found an increase in cup to disc ratios in addition to the deficits in nerve fiber layers. If intraocular pressure is the only criteria utilized to rule out glaucoma in studies, this may result in the inclusion of a variety of glaucomas: normal tension glaucomas, glaucomas that have a wide fluctuation in pressure, and glaucomas that had at baseline/young age very low pressures that are now within normal, but high normal ranges. An increased cup to disc ratio, also called cupping, is the physical phenomenon hypothesized to be caused by pressure at the posterior of the eye upon the nerve resulting in cell death and subsequently loss of the fibers themselves. The nerve has fewer nerve fibers along the edge or rim of the optic nerve. As more and more fibers are lost, the nerve starts to be hollowed out and then flattened. Figure 1 shows OCT images of the cup to disc ratio in a normal age-matched control participant, a participant with AD, and a participant with Parkinson's disease as well as glaucoma. 

Using the older OCT technology, Paquet and colleagues [2] studied a group of participants with both MCI and AD and looked at 15 noncognitively impaired age-matched participants, 14 participants with mild AD, 12 participants with moderate AD, and 23 participants with mild cognitive impairment. They found that the nerve fiber layer was thinned in those with mild cognitive impairment, mild AD, and moderate to severe AD. They also did not find any significant difference between those with mild AD and mild cognitive impairment. Even the older OCT imaging technology with its lower resolution has the potential to identify biomarkers of disease prior to substantial impact on the cognitive functions currently used to identify Alzheimer's disease. 

The older OCT technology has also been reported to identify deficits in the nerve fiber layer of those with Parkinson's disease compared to age-matched controls [68–70].

## 7. Conclusion

The visual system provides opportunity to use noninvasive clinical tools to identify and monitor biomarkers of neurodegenerative diseases such as AD. The number of those with AD in the United States is approaching 5.1 million, and this number is expected to reach 13.2 million by the year 2050 [71]. AD is estimated to cost Americans $100 billion a year both in direct costs and indirect costs [72]. OCT imaging has demonstrated structural nerve fiber deficits in the eye in early Alzheimer's disease as well as Mild Cognitive Impairment. Imaging with faster technology with significantly higher resolution such as the Fourier/Spectral/High Resolution-OCT may prove to be an efficient, cost-effective means of identifying and monitoring biomarkers of early AD.
